# The primary fallopian tube carcinoma: a rare association with pelvic nodal tuberculosis

**DOI:** 10.11604/pamj.2017.28.163.1119

**Published:** 2017-10-19

**Authors:** Nisrine Mamouni, Hanane Saadi, Hinde belfatemi, Sanaa Erraghay, Chahrazade Bouchikhi, Abdelaziz Banani

**Affiliations:** 1 Department of Gynecology and Obstetrics I, University Hospital Hassan II, Fez, Morocco; 2 Department of Pathology- University Hospital Hassan II, Fez, Morocco

**Keywords:** Primary carcinoma of fallopian tube, peritoneal tuberculosis, treatment, prognosis

## Abstract

The primary carcinoma of fallopian tube is a rare entity. It represents 0.14 to 1.81% of genital cancers in women. It is a cancer of older women. Its association with tuberculosis is exceptional. We report a rare case of bilateral serous adenocarcinoma of the fallopian tube in a patient aged 42 years, multiparous, whose characteristic is the unexpected association with peritoneal tuberculosis.

## Introduction

The primary fallopian tube carcinoma is rare, it occurs in older women. Its association with tuberculosis is exceptional. We report here a rare case of bilateral fallopian adenocarcinoma, in association with unexpected nodal tuberculosis.

## Patient and observation

**Figure 1:**
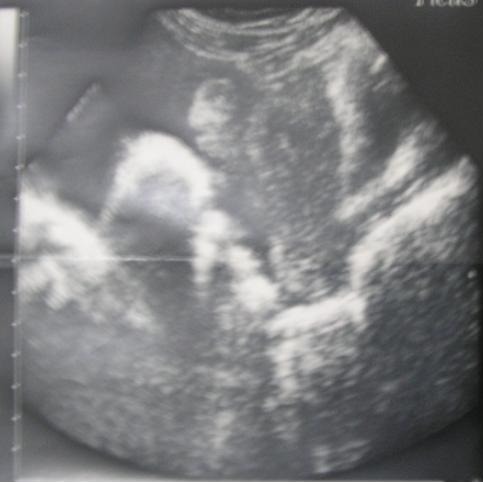
Abdominal pelvic ultrasound in longitudinal section Heterogeneous mass behind uterus with 4 centimeters of biggest diameter

**Figure 2:**
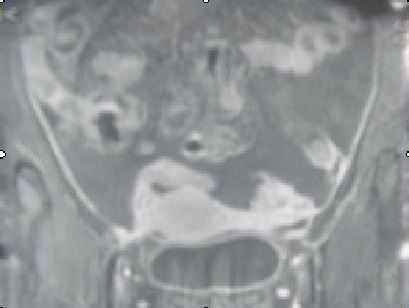
Pelvic MRI T2, image located in the left side of the uterus measuring 08 cm with dual component solid and cystic

**Figure 3:**
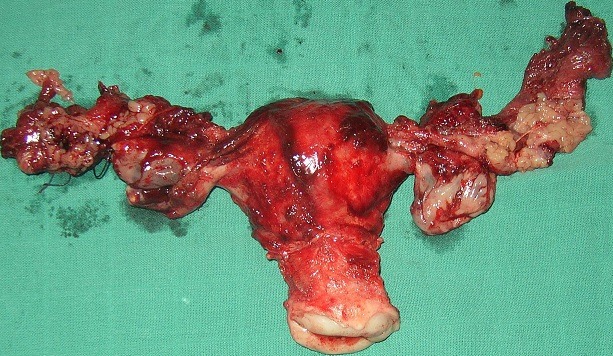
Surgical specimen

**Figure 4:**
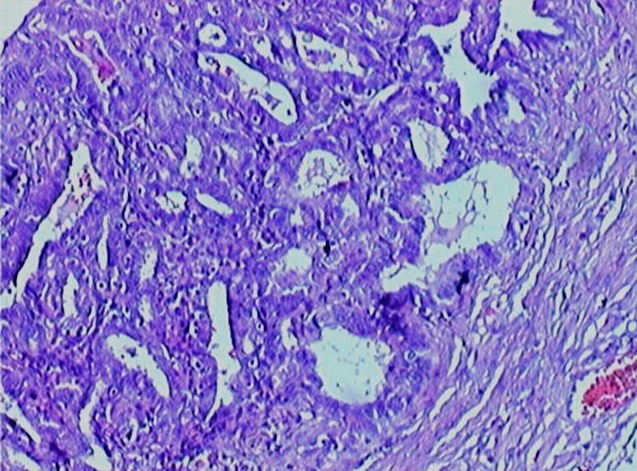
HES X 10, adenocarcinoma infiltrating the tubal wall until the sub serosa

A fourty two year old multiparous lady presented with pelvic pain four months before admission, radiating to the lumbar region without menstrual disorders without gastrointestinal symptoms or urinary symptoms. The clinical examination confirmed intraperitoneal effusion syndrome and found a pelvic mass located in the left side, mobilized from the uterus whose diameters are difficult to assess. The uterus is of normal size. Pelvic ultrasound showed an uterus of normal size with The presence an image located in the left side of the uterus, heterogeneous, large four centimetres of biggest diameter, a thick walled, taking the Doppler color at its periphery. The ovaries were not visualized (Figure 1). The magnetic imaging pelvic note the presence of ascites average abundance with an image located in the left side of the uterus measuring eight centimetres in long axis, with irregular contours, component solid and cystic. The left ovary is not seen and the right one is normal. The uterus is normal size and appearance (Figure 2). The biological assessment of the patient objectified that the determination of CA-125 increased. Laparotomy for suspected ovarian tumor was performed. The exploration intraoperative objective a citrine yellow ascites,with peritoneal millimiter granulations, the fallopian tubes were rigid seat of bilateral tumor. The uterus has a normal size; the left ovary is the sit of a cyst measuring three centimetres with thin wall, containing a liquid nutrunner.The surgical treatment consisted of a total hysterectomy with bilateral oophorectomy (Figure 3), omental and peritoneal biopsies and cytology collection of ascites. The histological examination was in favour of a serous adenocarcinoma of the two fallopian tubes, stage IIa according to the FIGO classification (Figure 4), infiltrating the right uterine horn. The ovaries and endometrium are not invaded. The biopsies showed omental and peritoneal granulomatous lesions. Absence of malignant cells in peritoneal fluid. The surgery was completed by bilateral pelvic lymph node dissection and omentectomy. The histological examination found the presence in lymph a tuberculous reaction with a caseous necrosis. An antibiotic treatment, based in streptomycin, Isoniazid, Rifampicin and Pirazinamide was administrated for one month and the cancer chemotherapy was started in parallel to the antibiotics. We administrated Cisplatin at a dose of 75mg/m^2^ and Adriablastin at a dose of 60mg/ m^²^. The patient was received four cycles of chemotherapy spaced 21 days apart. The patient was followed in consultation and assessment to the response to the treatment was based in clinical, biological (determination of CA -125) and CT scan. The patient was lost after the fourth cure of chemotherapy.

## Discussion

The primary cancer of fallopian tube is 0.14 to 1.81 % of all neoplasm of the femal genitalia [1]. According to Stewart [2], it occurs in postmenopausal women between 60 et 79 years, while for Kone [3], it is a disease that occurs readily during the fifth and sixth decade. However, our patient is young (42 years) and premenopausal. Several risk factors have been mentioned in connection with primary cancer of fallopian tube. The nulliparity is reported in 15% of cases in the series of Baalbaky [4], the association with a history of infertility found in 5 to 25% of cases according to Kone [3], and finally chronic tubal infection which is variously estimated [5, 6] because of some authors which considers that the bilateraty of salpingitis oppose the unilateralism of the fallopian tube carcinoma whose incidence appears to be much lower than that of salpingitis [7]. The combination of tubal carcinoma and genital tuberculosis is exceptional. Since 1950, we found in the literature eight cases of primary cancer of the fallopian tube associated with tuberculous salpingitis.The probability of a cause relationship has been raised but not confirmed because of the rarity of cases [8-14]. Clinically the primary carcinoma of the fallopian tube is manifested by hydrorrhoea in 61% of patients; it is often associated with bleeding [15]. Pelvic pain is inconstant and non-specific [16]. On physical exam, the pelvic mass is located in left side of the uterus, renitent, mobilized from the uterus, can be confused with ovarian mass [4]. Peroperative diagnosis is rare and often confused with ovarian and uterine pathology.

During the last decade, transvaginal pelvic ultrasound was able to make the diagnosis of processes of the fallopian tube in some cases. The pelvic scan and MRI, in addition to their interest in the diagnosis of tubal masses, they are recommended for the locoregional supervision under treatement, in association with tumor markers and CA125 [4]. The diagnosis is provided by the histological study and must meet the criteria of Hu and all [17]; the main tumor is located in the fallopian tube and develops from the tubal epithelium. The tumor contained tubal epithelial structure and there is a transition between normal tubal epithelium and invasive carcinoma. The uterus and the ovary contain only superficial cancerous formations. The therapeutic management of tubal cancer is similar of that of ovarian cancer [18], based on surgery to be as complete as possible: abdominal total hysterectomy, oophorectomy, omentectomy and peritoneal cytology [19]. The para-aortic and pelvic lymphadenectomy should be systematically given the frequency of nodal involvement even in early stages [19, 20]. Conserving surgery for patients wishing pregnancy may be considered for stage I. Postoperative adjuvant chemotherapy is indicated in case of infiltration of the mucosa or tumor rupture. The protocols based on Platinium and Adriamycin or Platinium and Taxanes have proven their effectiveness [20,21]. Our patient had the first protocol. Postoperative radiotherapy is not recommended. The hormone therapy is warranted given the response of the tubal epithelium to the hormones of menstrual cycle but must be codified [22]. The association with genital tuberculosis requires to disactivate the disease before starting immunosuppression by chemotherapy. Some authors proposed to administer the antibiotic treatment at least four weeks before the start of chemotherapy [9]. The prognosis of this neoplasm remains poor with a five years survival of 43% for all stages combined [23], the recurrences are common in the first three years [24] and the main prognostic factor is tumor stage [25].

## Conclusion

Primary carcinoma of the fallopian tube is a rare entity especially in young women. The preoperative diagnosis is difficult to establish. Its association with tuberculosis is even rarer and poses the problem of antibiotic treatment that must be started before chemotherapy.

## Competing interests

Tha authors declare no competing interests.
